# Symptomatic discoid lateral meniscus: a clinical and arthroscopic study in a Chinese population

**DOI:** 10.1186/s12891-016-1188-3

**Published:** 2016-08-05

**Authors:** Gang Chen, Zhong Zhang, Jian Li

**Affiliations:** Department of Orthopedic Surgery, West China Hospital, Sichuan University, No.37, Guoxue Alley, Chengdu, 610041 China

**Keywords:** Discoid lateral meniscus, Meniscus tear, Arthroscopy, Knee

## Abstract

**Background:**

Discoid lateral meniscus (DLM) is relatively common in East Asia..Symptomatic discoid lateral meniscus (SDLM) is an important indication for knee arthroscopic surgery. However, studies investigating SDLM are rare. The purpose of this study was to evaluate the clinical characteristics and intra-articular variants of SDLM in a Chinese population.

**Methods:**

We retrospectively reviewed all patients with SDLM from January 2005 to December 2014 in our hospital. Clinical variables included gender, age, duration, age of onset, affected side, symptoms and trauma history as well as arthroscopic findings: DLM types, tear patterns and concomitant medial meniscus tear, which were evaluated and compared statistically.

**Results:**

Of the 496 consecutive participants with SDLM, females outnumbered males (69.6 % vs. 30.4 %). The age of onset ranged from 3 to 80 years (median, 31 years), and was significantly higher in females than in males (*p* < 0.0001). Trauma history in males was significantly higher than in females (45 % vs. 35.1 %, *p* = 0.0356). Males showed a higher incidence of popping and snapping, while females manifested higher range-of-motion (ROM) limitations (*p* = 0.0179, and *p* = 0.0392, respectively). No significant difference in intra-articular variants was observed between genders. The complete type was the most frequent (344, 69.4 %), followed by the incomplete type (149, 30 %), and the Wrisberg type (3, 0.6 %). Significant difference in tear patterns was found between complete and incomplete types (*p* < 0.0001). Few patients showed medial meniscus tear (11, 2.2 %), at a significantly higher age compared with patients without tear (median, 57 years vs. 33 years, *p* < 0.0001).

**Conclusions:**

The majority of Chinese patients with SDLM are young and middle-aged females. Female patients had an older age of onset, higher incidence of ROM limitation and limited trauma history. The complete type is the most common, with tear patterns varying between complete and incomplete types. The SDLM does not significantly affect the medial meniscus.

## Background

Discoid lateral meniscus (DLM) is a variant of knee joint. It is relatively lower in incidence among Caucasians, but higher in East Asian races [1–6]. The etiology of DLM is still not very clear [6]. It is believed that congenital factors play an important role in DLM. Smillie [7] proposed the hypothesis of developmental stagnation, which has yet to be confirmed by embryological studies. A few studies [8] suggest that it is caused by acquired factors, which cannot fully explain the cause of DLM.

DLM associated with pain, popping and snapping, locking and other symptoms requiring medical intervention, is known as symptomatic discoid lateral meniscus (SDLM). SDLM may be due to the large and thick DLM, which blocks the natural contact of the lateral femoral condyle and tibial plateau, resulting in abnormal mechanical transduction. In addition, both disorganized collagen fibers and poor blood supply [9, 10] increase the risk of DLM rupture, leading to symptoms.

Most of the current studies in SDLM involve small sample sizes, lacking adequate description of the clinical characteristics, especially consensus on gender differences. Studies with a large sample size correlating the SDLM types and the tear patterns are unavailable. This study reviewed consecutive patients with SDLM in the past 10 years at one center, recorded and analyzed the clinical variables and arthroscopic findings, to identify the pattern of SDLM in Chinese.

## Methods

### Participant enrollment

In this retrospective study, consecutive patients with SDLM at the West China hospital, Sichuan University, were investigated from January 2005 to December 2014. The inclusion criteria were: (1) clear knee symptoms with the exception of other reasons; (2) DLM (intact or ruptured/torn) confirmed by MRI and arthroscopy; (3) presence or absence of a history of trauma, for instance, due to traffic accidents or sports injury; (4) and complete medical records. The exclusion criteria were: (1) ipsilateral limb deformity or dysfunction; (2) severe limb trauma, such as fracture, dislocation and ligament injury; (3) asymptomatic DLM discovered accidentally; (4) and incomplete medical records. We obtained approval from the Department of Orthopedic Surgery, West China Hospital, Sichuan University, to obtain access to the electrical medical records. In addition, this study was approved by the Institutional Review Board of West China Hospital, Sichuan University.

### Clinical study

The clinical variables including gender, age, duration, age of onset, affected side, symptoms and trauma history, were recorded. The age of onset was defined by symptom onset. In patients with bilateral symptoms, we recorded the age of initial involvement of the first affected side. According to age and symptom onset, we divided the patients into seven groups as follows: ages 0 to 9 years, group 1; 10 to 19 years, group 2; 20 to 29 years, group 3; 30 to 39 years, group 4; 40 to 49 years, group 5; 50 to 59 years, group 6; and 60 years and above under group 7. Based on monthly duration, we divided the patients into six groups: 0 to 9 months, group 1; 10 to 19 months, group 2; 20 to 29 months, group 3; 30 to 39 months, group 4; 40 to 49 months, group 5; and 50 months and older, group 6. According to the affected side, we recorded the left, right and bilateral symptoms. During the period covered by this review, if the symptoms involved both knees simultaneously or successively, we recorded them as bilateral. The symptoms were grouped into six categories, namely, pain, swelling, snapping and popping, locking, range-of-motion (ROM) limitation (mainly extension) and giving way. Trauma history was defined as knee trauma, such as sprain, tumbling and impact due to sports injuries or traffic accidents, except for fractures, dislocations and ligament injuries. According to patients’ complaints, we recorded the trauma history as positive or negative. For statistical reasons, we did not record the reasons and types of trauma. Further, we conducted a comparative analysis of the clinical variables to identify the SDLM features.

### Arthroscopy

After arthroscopy, the DLM types, tear patterns and combined injury were recorded. According to the Watanabe classification [11], the DLMs were divided into three categories: complete, incomplete and Wrisberg types. According to the Bin classification system [12], the DLM tears were divided into four categories: radial (including large flap tear), longitudinal (including bucket handle tear), horizontal and complex tears (with two or more types of tears, including severe degenerative tear). It was recorded as “no tear” if there was only simple degeneration or no obvious damage. Cases, which were difficult to classify, were reviewed by two experienced senior surgeons. Combined injury mainly referred to medial meniscus tear. We also analyzed the relationship between intra-articular variants as well as the relationship between arthroscopic findings and clinical variables.

### Data analysis

The quantitative indicators were not distributed normally, and therefore, we used the median, interquartile ranges as well as histograms. Further, we used nonparametric test (Wilcoxon rank sum test or Kruskal-Wallis H test) for analysis of the factors. Qualitative indices were analyzed by the Chi square test or Fisher's exact test. We used the SAS 9.4 (Institute of ASA, Cary, NC, USA). *p* < 0.05 was considered as statistically significant.

## Results

### Clinical research

**Fig. 1:**
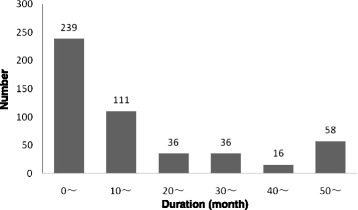
Distribution of duration

**Fig. 2:**
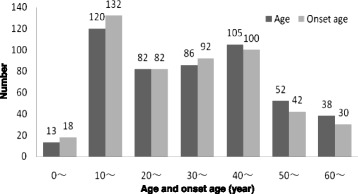
Age and onset age distribution

A total of 496 cases including 345 females (69.6 %) and 151 males (30.4 %) were studied. The patients manifested similar bilateral symptoms (Table 1). In addition, the duration, age and symptom onset were analyzed using the median and interquartile ranges (Table 2). The duration was within 10 months in 239 cases (48.2 %) and within 20 months in 350 cases (70.6 %) (Fig. 1).The majority of patients were young and middle-aged and included a wide age span: 393 (79.2 %) patients were aged between 10 and 49 years. The age of onset also demonstrated similar central tendency, including 406 cases (81.9 %) under the age span of 10 to 49 years (Fig. 2).

**Table 1:** Gender distribution of clinical variables

Variables		Total	Gender		*p*- value
			Female	Male	
Sides	Left	218 (44)	157 (45.5)	61 (40.4)	0.5725^a^
	Right	229 (46.1)	155 (44.9)	74 (49)	
	Both	49 (9.9)	33 (9.6)	16 (10.6)	
Trauma	Yes	189 (38.1)	121 (35.1)	68 (45)	0.0356^a*^
	No	307 (61.9)	224 (64.9)	83 (55)	
Symptom	Pain	477 (96.2)	330 (95.7)	147 (97.4)	0.1437^b^
	Swelling	135 (27.2)	95 (27.5)	40 (26.5)	0.0852^b^
	Popping & Snapping	166 (33.5)	107 (31)	59 (39.1)	0.0179^b*^
	Locking	79 (15.9)	55 (15.9)	24 (15.9)	0.1060^b^
	ROM limitation	114 (23)	85 (24.6)	29 (19.2)	0.0392^b*^
	Giving way	43 (8.7)	31 (9)	12 (7.9)	0.1307^b^

Values are presented as *n* (%)

^a^ *χ* ^2^ test

^b^Fisher exact test

^*^ *p* < 0.05

**Table 2:** Duration, age and onset age

Items	Range	Median	Range interquartile
Duration (month)	0–300	10	21
Age (year)	4–81	34	25
Onset age (year)	3–80	31	26

In this study, most of the patients lacked trauma history (Table 1). Based on the trauma history, the patients were classified. Wilcoxon rank sum test was used to compare the age of onset. The results showed no significant differences between groups (*p* = 0.2155). However, significant gender differences existed in trauma history (*p* < 0.05). Males showed a higher incidence of trauma history than females (Table 1).

In all the six categories, pain was the most common, followed in turn by popping and snapping, swelling, ROM limitation, locking and giving way. We found that male patients manifested a higher frequency of popping and snapping, while female patients had a higher incidence of ROM limitation, and the differences were significant (*p* < 0.05) (Table 1).

Symptom analysis of single patients revealed maximum number of cases with two symptoms (187, 37.7 %), followed by cases with one symptom (164, 33.1 %), and three symptoms (112, 22.6 %). In addition, cases with four or more symptoms occurred in the descending order, respectively: 26 cases (5.2 %), six cases (1.2 %) and one case (0.2 %).

Each individual symptom was assigned one point. We divided groups according to the trauma history, and performed the Wilcoxon rank sum test. Among different groups of trauma history, the scores were not significantly different (*p* = 0.1926).

Wilcoxon rank sum test showed significant differences in the age of onset between different genders (*p* < 0.0001). The concentration and dispersion were further analyzed. We found that the onset age of females was higher than in males.

### Arthroscopy

All patients underwent arthroscopy. The complete type of SDLM was the most common followed by the incomplete type, while the Wrisberg type was relatively rare. The longitudinal tear was the most common pattern followed by horizontal and complex tears. Radial tear was relatively infrequent. In addition, a small amount of SDLM patients showed no evident meniscus tear. Further, in this study, a very small number of patients (11, 2 %) showed medial meniscus injury. No gender-based differences were found between the groups in terms of tear patterns and medial meniscus injury (*p* > 0.05) (Table 3).

**Table 3:** Gender distribution of intra-articular variants

Variants		Total	Gender		*p*- value
			Female	Male	
Classification of DLM	Complete	344 (69.4)	235 (68.1)	109 (72.2)	0.5961^b^
	Incomplete	149 (30)	108 (31.3)	41 (27.1)	
	Wrisberg	3 (0.6)	2 (0.6)	1 (0.7)	
	Radial	62 (12.5)	44 (12.8)	18 (11.9)	0.5520^a^
	Horizontal	135 (27.2)	91 (26.4)	44 (29.14)	
Tear pattern	Longitudinal	170 (34.3)	123 (35.6)	47 (31.13)	
	Complex	99 (20)	68 (19.7)	31 (20.53)	
	No tear	30 (6)	19 (5.5)	11 (7.28)	
Medial meniscus	Tear	11 (2.2)	5 (1.4)	6 (4)	0.0578^b^
	No tear	485 (97.8)	340 (98.6)	145 (96)	

Values are presented as *n* (%)

^a^ *χ* ^2^ test

^b^Fisher exact test

The age of onset in different types of SDLM was compared using the Wilcoxon rank sum test, and the difference was not significant (*p* = 0.3512). The tear patterns of complete and incomplete types were compared and significant differences were found (*p* < 0.05). The three most common tear patterns of the incomplete type included longitudinal, radial and complex tears. The three most common tear patterns of the complete type included horizontal, longitudinal and complex tears. In addition, the specific correlations between the tear patterns are listed in Table 4.

**Table 4:** Comparison of tear patterns: complete vs. incomplete types

Types	Radial	Horizontal	Longitudinal	Complex	No tear	*p*- value	Coefficient of association
Complete	20 (5.8)	118 (34.3)	107 (31.1)	82 (23.9)	17 (4.9)	<0.0001^a*^	0.38
Incomplete	42 (28.2)	15 (10.1)	62 (41.6)	17 (11.4)	13 (8.7)		

Values are presented as *n* (%)

^a^ *χ* ^2^ test

^*^ *p* < 0.05

We divided groups according to tear patterns and compared the duration and symptom scores. Kruskal-Wallis H test revealed insignificant differences among the groups (*P* = 0.3606, *P* = 0.4621, respectively). Similarly, we divided groups according to the types of DLM and analyzed the differences in symptom scores using Kruskal-Wallis H test. The differences among the groups were insignificant (*p* = 0.1200).

We divided groups according to the medial meniscus injury. The age was compared using the Wilcoxon rank sum test, which revealed a significant difference between groups (*p* < 0.0001). We analyzed the tendency and dispersion and found that the patients with medial meniscus tear were significantly older than those without tears. Further, using the Wilcoxon rank sum test, we found insignificant differences in duration between the two groups (*p* = 0.6676). Finally, trauma history was compared using the Fisher’s exact test, which also revealed insignificant differences between the groups (*p* = 0.7550).

## Discussion

Significant differences exist in the incidence of DLM among different races increasing from Caucasians to the East Asian races [1–6]. Currently, no accurate epidemiological data are available for DLM. However, studies show great differences in design methods including cadaveric study, and clinical studies across specific age groups such as children or adults [6]. Clinical studies involving full age with a large sample size are unavailable. Further, although gender differences in DLM are described, no in-depth analysis is available. To the best of our knowledge, this is a retrospective study with the largest sample size of all age groups based on consecutive patients. Further, all the cases were diagnosed with MRI and arthroscopy, which revealed the shape, dimension and tear patterns accurately and comprehensively.

In this study, females with SDLM significantly outnumbered males, which was consistent with the studies of Papadopoulos [13] and Ahn [14]. The age of onset mainly focused on the age interval from 10 to 49 years, including youth and middle-aged groups. An obvious peak occurred in the interval between 10 and 19 years. The incidence tended to be stable from adulthood to old age. In children aged less than 10 years and in the elderly over 50 years, relatively few symptoms were seen suggesting that the symptoms were age-related. In youth, with rapid increase in movement, a peak was observed. In contrast, in adulthood with relatively stable exercise, the incidence was also relatively stable. Meanwhile, the age of onset in women was higher than in men, which may be specifically related to differences in intensity of exercise. For example, vigorous exercise in males during adolescence may lead to earlier symptom manifestation than in women.

In terms of the affected side, the two sides of knees showed similar incidence of symptoms. About 10 % of patients during the time period covered by this review manifested bilateral knee symptoms simultaneously or successively. Patients with unilateral complete SDLM had a probability of 89.5 % of complete DLM in contralateral knee [15]. In another study [16], 79 % of the patients with unilateral SDLM had contralateral DLM. Liu et al. [17] reported that 85 % of bilateral DLM manifested homotype. However, bilateral DLM was often observed with only unilateral occurrence of symptoms [4]. Our results showed that only a small proportion of the patients with unilateral SDLM manifested bilateral symptoms in 10 years.

This study also found that only a third of all the patients with SDLM reported exact trauma history, which was consistent with the study of Ellis [18]. It suggested the absence of any causal relation between symptoms and trauma. Except for trauma, the quality of decline in DLM may cause meniscus tear. In addition to the morphological abnormality in DLM, the histological disorder increases the susceptibility to shear force. In normal activities, the tear of the abnormal meniscus may also occur. Further, the incidence of trauma history in male patients was higher than in the female patients, due to the relatively large degree of exercise that might cause sports injury.

Compared with normal meniscus tear, no specific symptoms of DLM exist. Most patients exhibited one to three symptoms. The two most common symptoms were pain, and popping and snapping, which were similar to other studies [13, 19, 20]. Male patients showed a higher incidence of popping and snapping, while women manifested a higher ROM limitation. There was no significant difference in symptoms according to trauma history, suggesting that symptoms were mainly caused by the structure or tear of the meniscus. Further analysis showed no significant differences in symptom scores among DLM types as well as tear patterns, indicating that the symptoms were independent of specific DLM type or tear pattern.

The complete, incomplete and Wrisberg types of DLM were defined in relevant studies [3, 21]. In SDLM, the complete type was the predominant, followed by incomplete and Wrisberg types. The incomplete type outnumbered the complete type in Japanese populations [4]. However, the study used cadavers rather than patients and excluded patient symptoms resulting in bias. In clinical studies involving SDLM [13, 14], complete DLM was higher than the incomplete type, possibly due to the larger effects of the complete DLM on mechanical transmission of knee joint forces resulting in a higher risk of tear and symptoms. The incidence of Wrisberg type was approximately 0.2 % [22], which was similar to our study. No significant differences in the age of onset were found among the different DLM types.

DLM is susceptible to tear at a higher rate than the normal meniscus [23]. We found that the most common tear patterns of incomplete type were longitudinal and radial tears, while the most common tear patterns of complete type were horizontal and longitudinal tear. Kato [4] and Bin [12] reported similar discoveries. In incomplete DLM, the upper and the lower edges of the meniscus are not parallel. The profiles are more wedge-shaped, which is similar to amplified meniscus, leading to longitudinal and radial tears under shear force; In complete DLM, the upper and the lower edges are parallel, and the profiles are more rectangular, with a disordered internal fiber structure, resulting in lamellar tear under rolling action. A relatively large number of complex tears occur in complete and incomplete DLM, which may be derived from a single tear pattern under long-term effect of shear force. Notably, in this study, we also observed both complete and incomplete SDLM with no definite tear. The occurrence of symptoms in these patients may be caused by the abnormal shape of the meniscus itself or instability.

We also found a few patients with SDLM complicated with medial meniscus tear, which was unrelated with trauma history or duration, but had a relatively higher age, suggesting a degenerative lesion caused by increased age, with no direct relationship with DLM. Fukuta [5] and Rohren [23] reported changes in medial meniscus in patients with DLM that were not apparent in MRI. In addition, varus knees were relatively more common in patients with DLM than in normal people [24]. To our knowledge, the varus knee may lead to increased medial compartmental pressure, leading to medial meniscus tear, which was not consistent with our findings. In DLM, mechanical transduction is not similar to that of normal knees. In addition, the lower age of the population may raise the risk of degenerative medial meniscus tear.

The biggest advantage of this study is the large sample size with long time span, which allows sub-categorization of the patients with SDLM. Further, all the cases undergoing arthroscopy enabled investigation of intra-articular variants. However, the study limitations are as follows. First, this is a retrospective cohort study involving single center, and the results are applicable to the SDLM in East Asians, especially Chinese, and cannot be generalized to the whole population. Second, the duration and symptoms were totally reported by the patients themselves, which may affect the accuracy. Third, in a few patients with chronic conditions, the lateral meniscus was nearly damaged, and the type of DLM was only decided by two experienced doctors according to the imaging data (mainly MRIs) and surgical records. The tear pattern was complex, which might result in bias.

## Conclusions

In the Chinese, DLM, mainly in young and middle age, is associated with wide-ranging age-related symptoms. The patients were predominantly females. In addition, earlier manifestation in males than in females has been reported. Pain is the most common symptom in SDLM. Differences in symptoms between males and females were observed. The complete type is the most common. Different types of DLM show varied tear patterns. SDLM has less influence on medial meniscus.

## Abbreviations

DLM, discoid lateral meniscus; ROM, range of motion; SDLM, symptomatic discoid lateral meniscus

## References

[1] Vandermeer RD, Cunningham FK (1989). Arthroscopic treatment of the discoid lateral meniscus: results of long-term follow-up. Arthroscopy.

[2] Kim SJ, Lee YT, Kim DW (1998). Intraarticular anatomic variants associated with discoid meniscus in Koreans. Clin Orthop Relat Res.

[3] Ikeuchi H (1982). Arthroscopic treatment of the discoid lateral meniscus. Technique and long-term results. Clin Orthop Relat Res.

[4] Kato Y, Oshida M, Aizawa S, Saito A, Ryu J (2004). Discoid lateral menisci in Japanese cadaver knees. Mod Rheumatol.

[5] Fukuta S, Masaki K, Korai F (2002). Prevalence of abnormal findings in magnetic resonance images of asymptomatic knees. J Orthop Sci.

[6] Sun Y, Jiang Q (2011). Review of discoid meniscus. Orthop Surg.

[7] Smillie IS (1948). The congenital discoid meniscus. J Bone Joint Surg (Br).

[8] Bisicchia S, Tudisco C (2013). Re-growth of an incomplete discoid lateral meniscus after arthroscopic partial resection in an 11 year-old boy: a case report. BMC Musculoskelet Disord.

[9] Atay OA, Pekmezci M, Doral MN, Sargon MF, Ayvaz M, Johnson DL (2007). Discoid meniscus: An ultrastructural study with transmission electron microscopy. Am J Sports Med.

[10] Good CR, Green DW, Griffith MH (2007). Arthroscopic treatment of symptomatic discoid meniscus in children: classification, technique, and results. Arthroscopy.

[11] Watanabe M, Takeda S, Ikeuchi H (1979). Atlas of arthroscopy.

[12] Bin SI, Kim JC, Kim JM, Park SS, Han YK (2002). Correlation between type of discoid lateral menisci and tear pattern. Knee Surg Sports Traumatol Arthrosc.

[13] Papadopoulos A, Karathanasis A, Kirkos JM, Kapetanos GA (2009). Epidemiologic, clinical and arthroscopic study of the discoid meniscus variant in Greek population. Knee Surg Sports Traumatol Arthrosc.

[14] Ahn JH, Choi SH, Lee YS, Yoo JC, Chang MJ, Bae S (2011). Symptomatic torn discoid lateral meniscus in adults. Knee Surg Sports Traumatol Arthrosc.

[15] Chung JY, Roh JH, Kim JH, Kim JJ, Min BH (2015). Bilateral occurrence and morphologic analysis of complete discoid lateral meniscus. Yonsei Med J.

[16] Bae JH, Lim HC, Hwang DH, Song JK, Byun JS, Nha KW (2012). Incidence of bilateral discoid lateral meniscus in an Asian population: an arthroscopic assessment of contralateral knees. Arthroscopy.

[17] Liu WX, Zhao JZ, Huangfu XQ, He YH, Yang XG (2015). Prevalence of bilateral Discoid Lateral Menisci (DLM) in patients operated for symptomatic DLM with a follow-up study on their asymptomatic contralateral knees: a Magnetic Resonance Imaging (MRI) assessment. BMC Musculoskelet Disord.

[18] Ellis HB, Wise K, LaMont L, Copley L, Wilson P (2015). Prevalence of discoid meniscus during arthroscopy for isolated lateral meniscal pathology in the pediatric population. J Pediatr Orthop.

[19] Rao PS, Rao SK, Paul R (2001). Clinical, radiologic, and arthroscopic assessment of discoid lateral meniscus. Arthroscopy.

[20] Mutlu S, Mutlu H, Mutlu B, Guler O, Duymus TM (2014). Symptoms of discoid lateral menisci. J Orthop.

[21] Woods GW, Whelan JM (1990). Discoid meniscus. Clin Sports Med.

[22] Neuschwander DC, Drez D, Finney TP (1992). Lateral meniscal variant with absence of the posterior coronary ligament. J Bone Joint Surg Am.

[23] Rohren EM, Kosarek FJ, Helms CA (2001). Discoid lateral meniscus and the frequency of meniscal tears. Skeletal Radiol.

[24] Kim SJ, Bae JH, Lim HC (2013). Does torn discoid meniscus have effects on limb alignment and arthritic change in middle-aged patients?. J Bone Joint Surg Am.

